# (*Z*)-3-(2-Hy­droxy­eth­yl)-2-(phenyl­imino)-1,3-thia­zolidin-4-one

**DOI:** 10.1107/S1600536812030243

**Published:** 2012-07-07

**Authors:** Shaaban K. Mohamed, Antar A. Abdelhamid, Sabry H. H. Younes, Mahmoud A. A. Elremaily, Jim Simpson

**Affiliations:** a Chemistry and Environmental Division, Manchester Metropolitan University, Manchester M1 5GD, England; b Chemistry Department, Faculty of Science, Sohag University, Sohag 82524, Egypt; c Department of Chemistry, University of Otago, PO Box 56, Dunedin, New Zealand

## Abstract

In the title compound, C_11_H_12_N_2_O_2_S, the thia­zole and phenyl rings are inclined at 56.99 (6)° to one another. The thia­zole ring is planar with an r.m.s. deviation for the five ring atoms of 0.0274 Å. The presence of the phenyl­imine substituent is confirmed with the C=N distance to the thia­zole ring of 1.2638 (19) Å. The mol­ecule adopts a Z conformation with respect to this bond. The –OH group of the hy­droxy­ethyl substituent is disordered over two positions with relative occupancies 0.517 (4) and 0.483 (4). In the crystal, O—H⋯O hydrogen bonds, augmented by C—H⋯N contacts, form dimers with *R*_2_^2^(11) rings and generate chains along the *b* axis. Parallel chains are linked in an obverse fashion by weak C—H⋯S hydrogen bonds. C—H⋯O hydrogen bonds together with C—H⋯π contacts further consolidate the structure, stacking mol­ecules along the *b* axis.

## Related literature

For pharmaceutical background to thia­zolidinone compounds, see: Shah & Desai (2007 ▶); Subudhi *et al.* (2007 ▶); Kuecuekguezel *et al.* (2006 ▶); Mehta *et al.* (2006 ▶); Srivastava *et al.* (2006 ▶); Zhou *et al.* (2008 ▶). For our recent work on the synthesis of bio-selective mol­ecules, see: Mohamed *et al.* (2012 ▶). For related structures, see: Bally & Mornon (1973 ▶); Moghaddam & Hojabri (2007 ▶); Yella *et al.* (2008 ▶); Abdel-Aziz *et al.* (2012 ▶). For standard bond distances, see: Allen *et al.* (1987 ▶). For hydrogen-bond motifs, see: Bernstein *et al.* (1995 ▶).
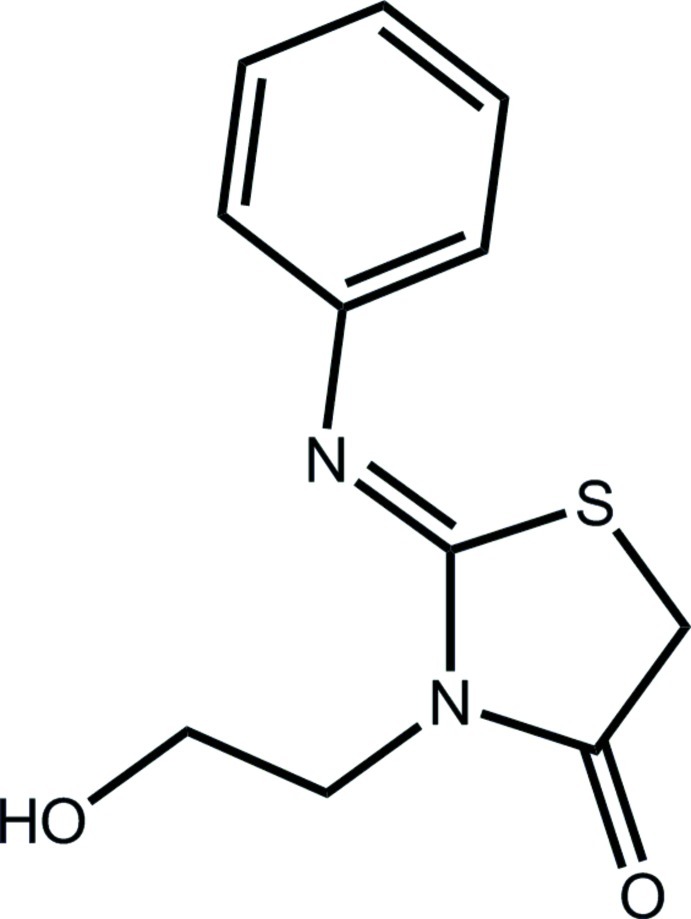


## Experimental

### 

#### Crystal data

C_11_H_12_N_2_O_2_S
*M* *_r_* = 236.29
Monoclinic, 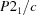

*a* = 11.9612 (6) Å
*b* = 6.9478 (3) Å
*c* = 13.1554 (6) Å
β = 91.244 (2)°
*V* = 1093.01 (9) Å^3^
*Z* = 4
Mo *K*α radiation
μ = 0.28 mm^−1^
*T* = 91 K
0.40 × 0.26 × 0.11 mm

#### Data collection

Bruker APEXII CCD area-detector diffractometer
Absorption correction: multi-scan (*SADABS*; Bruker, 2011 ▶) *T*_min_ = 0.693, *T*_max_ = 0.746
17811 measured reflections
2547 independent reflections
2150 reflections with *I* > 2σ(*I*)
*R*_int_ = 0.038

#### Refinement

*R*[*F* ^2^ > 2σ(*F* ^2^)] = 0.041
*wR*(*F* ^2^) = 0.100
*S* = 1.08
2547 reflections
157 parameters
6 restraints
H-atom parameters constrained
Δρ_max_ = 0.79 e Å^−3^
Δρ_min_ = −0.68 e Å^−3^

### 

Data collection: *APEX2* (Bruker, 2011 ▶); cell refinement: *APEX2* and *SAINT* (Bruker, 2011 ▶); data reduction: *SAINT*; program(s) used to solve structure: *SHELXS97* (Sheldrick, 2008 ▶) and *TITAN* (Hunter & Simpson, 1999 ▶); program(s) used to refine structure: *SHELXL97* (Sheldrick, 2008 ▶) and *TITAN*; molecular graphics: *SHELXTL* (Sheldrick, 2008 ▶) and *Mercury* (Macrae *et al.*, 2008 ▶); software used to prepare material for publication: *SHELXL97*, *enCIFer* (Allen *et al.*, 2004 ▶), *PLATON* (Spek, 2009 ▶) and *publCIF* (Westrip, 2010 ▶).

## Supplementary Material

Crystal structure: contains datablock(s) global, I. DOI: 10.1107/S1600536812030243/tk5126sup1.cif

Structure factors: contains datablock(s) I. DOI: 10.1107/S1600536812030243/tk5126Isup2.hkl

Supplementary material file. DOI: 10.1107/S1600536812030243/tk5126Isup3.cml

Additional supplementary materials:  crystallographic information; 3D view; checkCIF report

## Figures and Tables

**Table 1:** Hydrogen-bond geometry (Å, °) *Cg*2 is the centroid of the C6–C11 phenyl ring.

*D*—H⋯*A*	*D*—H	H⋯*A*	*D*⋯*A*	*D*—H⋯*A*
O2—H2⋯O1^i^	0.84	1.98	2.802 (3)	168
C13—H13*B*⋯O1^ii^	0.99	2.67	3.407 (3)	131
C1—H1*A*⋯O1^iii^	0.99	2.56	3.472 (3)	153
C12—H12*B*⋯S1^iv^	0.99	2.92	3.613 (2)	128
C1—H1*B*⋯N5^v^	0.99	2.57	3.519 (3)	162
C9—H9⋯*Cg*2^vi^	0.95	2.77	3.5731 (16)	142

Symmetry codes: (i) 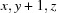; (ii) 
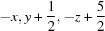
; (iii) 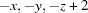; (iv) 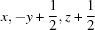; (v) 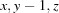; (vi) 
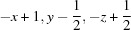
.

**Fig. 1.**
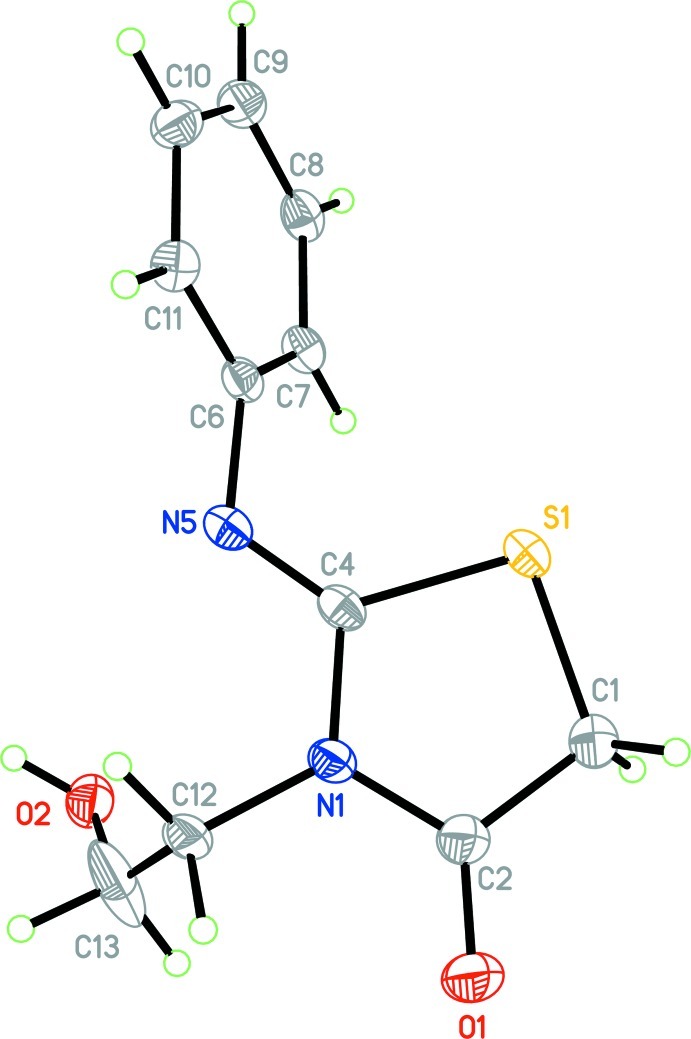
The structure of I with ellipsoids drawn at the 50% probability level. Only the major disorder component is shown.

**Fig. 2.**
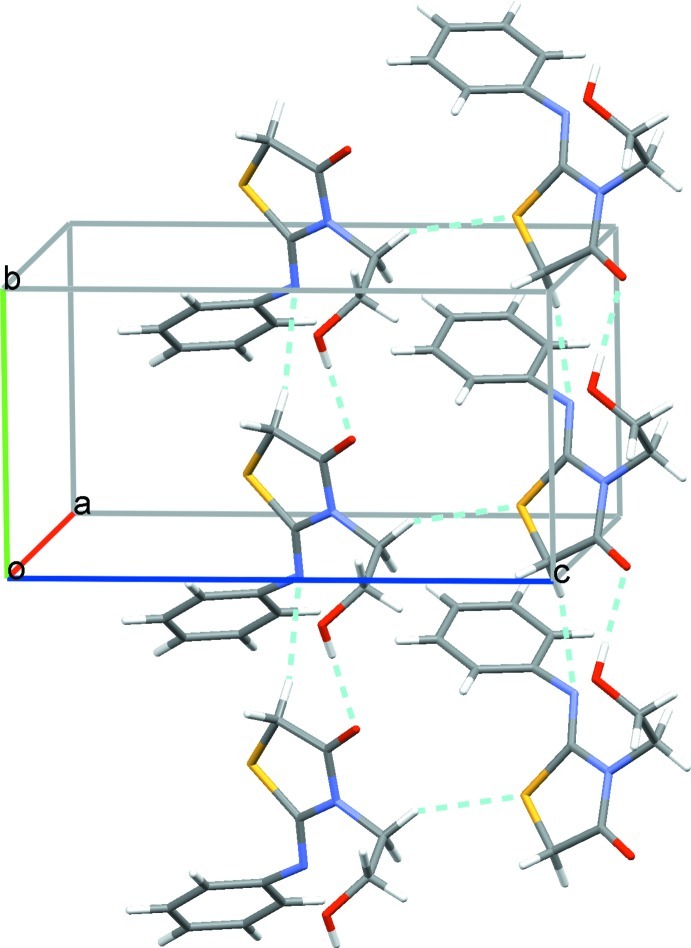
A view of the packing along the a axis showing chains of molecules linked by C–H···S hydrogen bonds. Hydrogen bonds are drawn as dashed lines and only the major disorder component is shown.

**Fig. 3.**
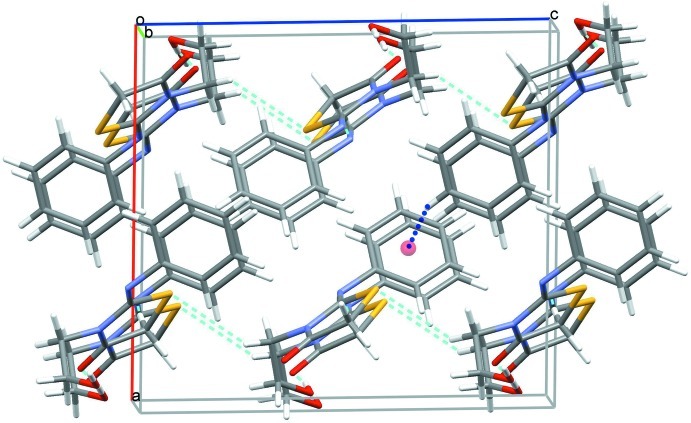
Overall packing for (1) viewed along the b axis showing a representative C–H···π contact as a dotted line. The red sphere represents the centroid of the C6···C11 phenyl ring. Hydrogen bonds are drawn as dashed lines and only the major disorder component is shown.

## supplementary crystallographic information

### Comment

Compounds incorporating the thiazolidinone core structure are of great interest to chemists and biologists due to their extensive bioactivities (Shah & Desai, 2007). These include anti-microbial (Subudhi *et al.*, 2007), anti-mycobacterial (Kuecuekguezel *et al.*, 2006), anti-inflammatory (Srivastava *et al.*, 2006), anti-fungal (Mehta *et al.*, 2006) and anti-cancer effects (Zhou *et al.*, 2008). In this context and following our on-going study of the synthesis of bio-selective molecules we were interested in investigating the microbial inhibiting effect of a newly synthesized series of compounds incorporating thiazolidinone ring systems. The synthesis of such compounds was carried out *via* a three component reaction technique using amino alcohols as precursors (Mohamed *et al.*, 2012). In this study, the crystal structure determination of the title compound (I) was undertaken to investigate the relationship between its structure and anti-bacterial activity.

The title compound (I), a phenylimino-thiazolidinone derivative, crystallizes with the S1/C1/C2/N1/C4 thiazole and C6···C11 phenyl rings inclined at 56.99 (6) ° to one another. The thiazole ring is planar with an r.m.s. deviation for the five ring atoms of 0.0274 Å. The C4═N5 distance, 1.2638 (19) Å, confirms this as a double bond and the molecule adopts a *Z* conformation with respect to this bond. The OH group of the hydroxyethyl substituent is disordered over two positions with relative occupancies 0.517 (4) for O2–H2 and 0.483 (4) for O3–H3. Bond distances (Allen *et al.*, 1987) and angles in the molecule are normal and similar to those found in related structures (Bally & Mornon, 1973; Moghaddam & Hojabri, 2007; Yella *et al.*, 2008; Abdel-Aziz *et al.*, 2012).

In the crystal structure head to tail dimers are formed from O2–H2···O1 hydrogen bonds, bolstered by weaker C1–H1B···N1 interactions, Table 1, forming *R*^2^_2_(11) rings (Bernstein *et al.*, 1995). These also link pairs of molecules into chains along *b*. Weak C12–H1B···S1 contacts join each chain to an equivalent one progressing in the opposite direction, Fig. 2. Two additional C–H···O hydrogen bonds together with C9–H9···π contacts further consolidate the structure forming stacks along *b*, Fig. 3.

### Experimental

To a well stirred mixture of 135 mg (1 mmol) phenylisothiocyanate and 61 mg (1 mmol) 2-aminoethanol in 50 ml dioxane, 167 mg (1 mmol) of bromo ethylacetate was added. The reaction mixture was refluxed and monitored by TLC until completion after 3 h. A solid product was deposited on cooling to room temperature and collected by filtration. The crude product was recrystallized from ethanol to give a high quality crystals (*M*.p. 327 K) suitable for X-ray analysis in an excellent yield (92%).

### Refinement

The OH group of the hydroxyethyl substituent is disordered over two positions O2 and O3 with relative occupancies that converged to 0.517 (4) and 0.483 (4). Displacement parameters for the C13 atom bound to the disordered OH groups were slightly higher than normal but a suitable additional disorder model could not be found. All H-atoms bound to carbon were refined using a riding model with d(C—H) = 0.95 Å for aromatic and 0.99 Å for CH_2_ H atoms, and with *U*_iso_ = 1.2*U*_eq_ (C). For the disordered O—H atoms d(O—H) = 0.84 Å, with *U*_iso_ = 1.5*U*_eq_ (O).

### Crystal data

**Table:**

C_11_H_12_N_2_O_2_S	*F*(000) = 496
*M**_r_* = 236.29	*D*_x_ = 1.436 Mg m^−^^3^
Monoclinic, *P*2_1_/*c*	Mo *K*α radiation, λ = 0.71073 Å
Hall symbol: -P 2ybc	Cell parameters from 5327 reflections
*a* = 11.9612 (6) Å	θ = 3.3–27.6°
*b* = 6.9478 (3) Å	µ = 0.28 mm^−^^1^
*c* = 13.1554 (6) Å	*T* = 91 K
β = 91.244 (2)°	Irregular block, yellow
*V* = 1093.01 (9) Å^3^	0.40 × 0.26 × 0.11 mm
*Z* = 4	

### Data collection

**Table:**

Bruker APEXII CCD area-detector diffractometer	2547 independent reflections
Radiation source: fine-focus sealed tube	2150 reflections with *I* > 2σ(*I*)
Graphite monochromator	*R*_int_ = 0.038
φ and ω scans	θ_max_ = 27.7°, θ_min_ = 3.1°
Absorption correction: multi-scan (*SADABS*; Bruker, 2011)	*h* = −15→15
*T*_min_ = 0.693, *T*_max_ = 0.746	*k* = −9→9
17811 measured reflections	*l* = −17→15

### Refinement

**Table:**

Refinement on *F*^2^	Primary atom site location: structure-invariant direct methods
Least-squares matrix: full	Secondary atom site location: difference Fourier map
*R*[*F*^2^ > 2σ(*F*^2^)] = 0.041	Hydrogen site location: inferred from neighbouring sites
*wR*(*F*^2^) = 0.100	H-atom parameters constrained
*S* = 1.08	*w* = 1/[σ^2^(*F*_o_^2^) + (0.0373*P*)^2^ + 0.9645*P*] where *P* = (*F*_o_^2^ + 2*F*_c_^2^)/3
2547 reflections	(Δ/σ)_max_ < 0.001
157 parameters	Δρ_max_ = 0.79 e Å^−^^3^
6 restraints	Δρ_min_ = −0.68 e Å^−^^3^

### Special details

**Table:**

Geometry. All e.s.d.'s (except the e.s.d. in the dihedral angle between two l.s. planes) are estimated using the full covariance matrix. The cell e.s.d.'s are taken into account individually in the estimation of e.s.d.'s in distances, angles and torsion angles; correlations between e.s.d.'s in cell parameters are only used when they are defined by crystal symmetry. An approximate (isotropic) treatment of cell e.s.d.'s is used for estimating e.s.d.'s involving l.s. planes.
Refinement. Refinement of *F*^2^ against ALL reflections. The weighted *R*-factor *wR* and goodness of fit *S* are based on *F*^2^, conventional *R*-factors *R* are based on *F*, with *F* set to zero for negative *F*^2^. The threshold expression of *F*^2^ > σ(*F*^2^) is used only for calculating *R*-factors(gt) *etc*. and is not relevant to the choice of reflections for refinement. *R*-factors based on *F*^2^ are statistically about twice as large as those based on *F*, and *R*- factors based on ALL data will be even larger.

### Fractional atomic coordinates and isotropic or equivalent isotropic displacement parameters (Å^2^)

**Table:**

	*x*	*y*	*z*	*U*_iso_*/*U*_eq_	Occ. (<1)
S1	0.27696 (4)	0.19764 (7)	0.90742 (4)	0.02132 (14)	
C1	0.18673 (18)	0.0254 (3)	0.96766 (16)	0.0261 (4)	
H1A	0.1232	−0.0083	0.9216	0.031*	
H1B	0.2287	−0.0937	0.9843	0.031*	
C2	0.14480 (17)	0.1172 (3)	1.06312 (15)	0.0248 (4)	
O1	0.08555 (15)	0.0341 (2)	1.12382 (12)	0.0393 (4)	
N1	0.17971 (13)	0.3030 (2)	1.07448 (12)	0.0204 (3)	
C4	0.24668 (14)	0.3775 (3)	0.99792 (13)	0.0172 (4)	
N5	0.28128 (12)	0.5494 (2)	1.00068 (11)	0.0180 (3)	
C6	0.34388 (14)	0.6235 (3)	0.91831 (14)	0.0171 (4)	
C7	0.29991 (15)	0.6270 (3)	0.81901 (14)	0.0196 (4)	
H7	0.2298	0.5682	0.8039	0.023*	
C8	0.35896 (16)	0.7167 (3)	0.74242 (15)	0.0213 (4)	
H8	0.3292	0.7179	0.6749	0.026*	
C9	0.46134 (17)	0.8049 (3)	0.76381 (15)	0.0234 (4)	
H9	0.5014	0.8661	0.7113	0.028*	
C10	0.50430 (16)	0.8025 (3)	0.86274 (15)	0.0233 (4)	
H10	0.5742	0.8623	0.8777	0.028*	
C11	0.44593 (15)	0.7135 (3)	0.94010 (14)	0.0201 (4)	
H11	0.4755	0.7139	1.0077	0.024*	
C12	0.14901 (17)	0.4171 (3)	1.16360 (15)	0.0255 (4)	
H12A	0.2113	0.5057	1.1812	0.031*	
H12B	0.1393	0.3292	1.2220	0.031*	
C13	0.0447 (2)	0.5319 (4)	1.14846 (19)	0.0439 (6)	
H13A	−0.0167	0.4364	1.1469	0.053*	
H13B	0.0363	0.6054	1.2123	0.053*	
O2	0.0188 (2)	0.6593 (4)	1.0724 (2)	0.0241 (8)	0.517 (4)
H2	0.0483	0.7663	1.0856	0.036*	0.517 (4)
O3	−0.0418 (2)	0.4527 (5)	1.1267 (2)	0.0316 (9)	0.483 (4)
H3	−0.0409	0.4168	1.0658	0.047*	0.483 (4)

### Atomic displacement parameters (Å^2^)

**Table:**

	*U*^11^	*U*^22^	*U*^33^	*U*^12^	*U*^13^	*U*^23^
S1	0.0221 (2)	0.0226 (3)	0.0195 (2)	0.00206 (18)	0.00538 (17)	−0.00315 (18)
C1	0.0326 (10)	0.0206 (10)	0.0254 (10)	−0.0003 (8)	0.0066 (8)	−0.0018 (8)
C2	0.0280 (10)	0.0244 (10)	0.0221 (10)	−0.0040 (8)	0.0038 (8)	−0.0013 (8)
O1	0.0548 (10)	0.0346 (9)	0.0292 (8)	−0.0195 (8)	0.0170 (7)	−0.0056 (7)
N1	0.0211 (8)	0.0236 (8)	0.0166 (8)	−0.0037 (6)	0.0043 (6)	−0.0034 (6)
C4	0.0140 (8)	0.0230 (9)	0.0146 (8)	0.0029 (7)	−0.0001 (6)	−0.0010 (7)
N5	0.0161 (7)	0.0229 (8)	0.0150 (7)	0.0014 (6)	0.0010 (6)	−0.0006 (6)
C6	0.0181 (8)	0.0163 (8)	0.0170 (9)	0.0037 (7)	0.0029 (7)	−0.0007 (7)
C7	0.0197 (8)	0.0203 (9)	0.0187 (9)	0.0040 (7)	0.0008 (7)	−0.0016 (7)
C8	0.0280 (9)	0.0186 (9)	0.0172 (9)	0.0058 (7)	0.0014 (7)	0.0014 (7)
C9	0.0302 (10)	0.0182 (9)	0.0220 (10)	0.0017 (8)	0.0066 (8)	0.0043 (8)
C10	0.0221 (9)	0.0205 (9)	0.0272 (10)	−0.0028 (7)	0.0018 (8)	0.0020 (8)
C11	0.0223 (9)	0.0191 (9)	0.0187 (9)	0.0009 (7)	−0.0014 (7)	0.0013 (7)
C12	0.0322 (10)	0.0290 (10)	0.0157 (9)	−0.0098 (8)	0.0084 (8)	−0.0067 (8)
C13	0.0564 (10)	0.0410 (10)	0.0346 (9)	0.0164 (8)	0.0045 (8)	−0.0037 (8)
O2	0.0295 (15)	0.0209 (14)	0.0217 (15)	−0.0021 (11)	−0.0001 (11)	−0.0017 (11)
O3	0.0208 (15)	0.054 (2)	0.0197 (16)	0.0020 (14)	0.0030 (11)	−0.0044 (15)

### Geometric parameters (Å, º)

**Table:**

S1—C4	1.7689 (19)	C8—H8	0.9500
S1—C1	1.806 (2)	C9—C10	1.389 (3)
C1—C2	1.504 (3)	C9—H9	0.9500
C1—H1A	0.9900	C10—C11	1.392 (3)
C1—H1B	0.9900	C10—H10	0.9500
C2—O1	1.224 (2)	C11—H11	0.9500
C2—N1	1.364 (3)	C12—C13	1.490 (3)
N1—C4	1.400 (2)	C12—H12A	0.9900
N1—C12	1.469 (2)	C12—H12B	0.9900
C4—N5	1.264 (2)	C13—O3	1.202 (4)
N5—C6	1.427 (2)	C13—O2	1.367 (4)
C6—C11	1.396 (3)	C13—H13A	0.9900
C6—C7	1.398 (3)	C13—H13B	0.9900
C7—C8	1.391 (3)	O2—H2	0.8400
C7—H7	0.9500	O3—H3	0.8400
C8—C9	1.393 (3)		
			
C4—S1—C1	92.29 (9)	C10—C9—C8	119.34 (18)
C2—C1—S1	107.38 (14)	C10—C9—H9	120.3
C2—C1—H1A	110.2	C8—C9—H9	120.3
S1—C1—H1A	110.2	C9—C10—C11	120.61 (18)
C2—C1—H1B	110.2	C9—C10—H10	119.7
S1—C1—H1B	110.2	C11—C10—H10	119.7
H1A—C1—H1B	108.5	C10—C11—C6	119.96 (17)
O1—C2—N1	123.72 (18)	C10—C11—H11	120.0
O1—C2—C1	123.58 (19)	C6—C11—H11	120.0
N1—C2—C1	112.69 (17)	N1—C12—C13	113.91 (18)
C2—N1—C4	116.73 (16)	N1—C12—H12A	108.8
C2—N1—C12	121.13 (16)	C13—C12—H12A	108.8
C4—N1—C12	122.13 (16)	N1—C12—H12B	108.8
N5—C4—N1	121.38 (16)	C13—C12—H12B	108.8
N5—C4—S1	127.96 (14)	H12A—C12—H12B	107.7
N1—C4—S1	110.59 (13)	O3—C13—O2	86.7 (3)
C4—N5—C6	119.74 (16)	O3—C13—C12	120.0 (3)
C11—C6—C7	119.60 (17)	O2—C13—C12	128.4 (2)
C11—C6—N5	118.48 (16)	O2—C13—H13A	105.2
C7—C6—N5	121.54 (16)	C12—C13—H13A	105.2
C8—C7—C6	119.88 (17)	O3—C13—H13B	109.6
C8—C7—H7	120.1	O2—C13—H13B	105.2
C6—C7—H7	120.1	C12—C13—H13B	105.2
C7—C8—C9	120.61 (18)	H13A—C13—H13B	105.9
C7—C8—H8	119.7	C13—O2—H2	109.5
C9—C8—H8	119.7	C13—O3—H3	109.5

### Hydrogen-bond geometry (Å, º)

Cg2 is the centroid of the C6–C11 phenyl ring.

**Table:**

*D*—H···*A*	*D*—H	H···*A*	*D*···*A*	*D*—H···*A*
O2—H2···O1^i^	0.84	1.98	2.802 (3)	168
C13—H13*B*···O1^ii^	0.99	2.67	3.407 (3)	131
C1—H1*A*···O1^iii^	0.99	2.56	3.472 (3)	153
C12—H12*B*···S1^iv^	0.99	2.92	3.613 (2)	128
C1—H1*B*···N5^v^	0.99	2.57	3.519 (3)	162
C9—H9···*Cg*2^vi^	0.95	2.77	3.5731 (16)	142

Symmetry codes: (i) *x*, *y*+1, *z*; (ii) −*x*, *y*+1/2, −*z*+5/2; (iii) −*x*, −*y*, −*z*+2; (iv) *x*, −*y*+1/2, *z*+1/2; (v) *x*, *y*−1, *z*; (vi) −*x*+1, *y*−1/2, −*z*+1/2.
